# Bio-electrosprayed human neural stem cells are viable and maintain their differentiation potential

**DOI:** 10.12688/f1000research.19901.2

**Published:** 2020-07-31

**Authors:** Citlali Helenes González, Suwan N. Jayasinghe, Patrizia Ferretti

**Affiliations:** 1 Stem Cell and Regenerative Medicine Section, UCL Great Ormond Street Institute of Child Health, University College London, London, WC1N 1EH, UK; 2 BioPhysics Group, Department of Mechanical Engineering, University College London, London, WC1E 7JE, UK

**Keywords:** bio-electrospray, death, differentiation, human, in vitro, neural stem cells, neurone, survival

## Abstract

**Background:** Bio-electrospray (BES) is a jet-based delivery system driven by an electric field that has the ability to form micro to nano-sized droplets. It holds great potential as a tissue engineering tool as it can be used to place cells into specific patterns. As the human central nervous system (CNS) cannot be studied *in vivo *at the cellular and molecular level, *in vitro* CNS models are needed. Human neural stem cells (hNSCs) are the CNS building block as they can generate both neurones and glial cells.

**Methods:** Here we assessed for the first time how hNSCs respond to BES. To this purpose, different hNSC lines were sprayed at 10 kV and their ability to survive, grow and differentiate was assessed at different time points.

**Results:** BES induced only a small and transient decrease in hNSC metabolic activity, from which the cells recovered by day 6, and no significant increase in cell death was observed, as assessed by flow cytometry. Furthermore, bio-electrosprayed hNSCs differentiated as efficiently as controls into neurones, astrocytes and oligodendrocytes, as shown by morphological, protein and gene expression analysis.

**Conclusions:** This study highlights the robustness of hNSCs and identifies BES as a suitable technology that could be developed for the direct deposition of these cells in specific locations and configurations.

## Introduction

Electrospraying is a very useful technique for fabricating micro- and nano-structures of different composition, texture and shape using a wide range of materials and cells. When cells are electrosprayed, the technique is known as bio-electrospray (BES). BES consists of a jet-based delivery system connected to an electric field that has the ability to generate nano-sized and cell-laden microdroplets ( Jayasinghe, 2011; Jayasinghe & Townsend-Nicholson, 2006; Poncelet *et al*., 2012). This is due to the difference in electric potential between the charged needle and the ground electrode that forms an electric field, accelerating the charged cell suspension within the needle and forming an unstable jet. This methodology has the advantage of having the potential to achieve single-cell delivery, giving a more homogeneous cell distribution within a 3-dimensional (3D) construct, as well as being very suitable for microencapsulation ( Boda *et al*., 2018; Jayasinghe, 2011; Poncelet *et al*., 2012). The configuration needed to obtain micro to nano-sized droplets and a near mono-distribution can be achieved by adjusting BES conditions to obtain a stable cone jet mode. The primary conditions to consider are viscosity and electrical conductivity of the sprayed liquid. Ideally, the cell-laden suspension should have high viscosity and low electrical conductivity. A coaxial arrangement with an outer needle carrying an encapsulating biomaterial and an inner needle carrying the cells in suspension could also be considered ( Jayasinghe *et al*., 2006; Jayasinghe & Townsend-Nicholson, 2006). Other strategies adopted to obtain such a high resolution require small diameter needles, resulting in shear stress to the cells and an inability to process high-density and/or viscous cell suspensions ( Greig & Jayasinghe, 2008; Hall *et al*., 2008).

An important consideration for cell-based applications is that the high voltages and spraying action could have an adverse effect on the cells, and this may differ among cell types. Although it has been demonstrated that BES does not significantly affect a range of mammalian cells, and even small organisms, its effect have never been studied on human neural stem cells (hNSCs), the building block of the nervous system ( Clarke & Jayasinghe, 2008; Geach *et al*., 2009; Hong *et al*., 2010; Jayasinghe *et al*., 2011; Joly *et al*., 2009; Tezera *et al*., 2017).

hNSCs either derived from the embryonic nervous system or differentiated from pluripotent stem cells provide an ideal source for modelling the human nervous system. hNSCs have the capacity to self-renew and differentiate into the major cell types of the brain, neurones and glia (oligodendrocytes and astrocytes), and hold the potential to repair damaged tissue in the central nervous system (CNS) ( Bianco & Robey, 2001; Gage & Temple, 2013). This makes them invaluable for the development of 3D models for the study of normal and abnormal developmental mechanisms, neurodegenerative disorders, neural repair and high-throughput screening of putative neuroactive drugs ( Breier *et al*., 2010; Gage & Temple, 2013; Gu *et al*., 2016). There is also much interest in using hNSC to develop 3D systems for transplantation into the damaged CNS ( Somaa *et al*., 2017; Vishwakarma *et al*., 2014).

Given the encouraging results from a few studies on mouse neural cells and human astrocytoma ( Eagles *et al*., 2006; Eddaoudi *et al*., 2010; Jayasinghe & Townsend-Nicholson, 2006; Mongkoldhumrongkul *et al*., 2009a), we wished to establish whether hNSCs could be bio-electrosprayed, and specifically assess whether the procedure affected their survival and ability to undergo multi-lineage differentiation. Extensive analysis of hNSC survival/death and differentiation showed that hNSCs withstand the BES procedure very well and could successfully differentiate towards neuronal, astrocyte and oligodendrocyte lineages with no alteration in gene expression following neuronal differentiation. Together, this study demonstrates that hNSCs remain viable over prolonged periods post-treatment and are capable of withstanding the pressure and stresses of being handled as high-density cell suspensions within a needle at a high voltage.

## Methods

### Materials

Unless otherwise indicated, chemicals were purchased from Sigma-Aldrich (UK). Dulbecco’s Modified Eagle Medium/Nutrient Mixture F-12 GlutaMAX ^TM^ (DMEM/F12, Thermo Fisher Scientific, 31331028), Neurobasal®-A Medium (Life Technologies, 10888022), foetal bovine serum (FBS, Life Technologies, 10270106), N-2 supplement 100x (Thermo Fisher Scientific, 17502048) and B-27 supplement 50x (Thermo Fisher Scientific, 12587010), human FGF-2 (Peprotech, AF-100-18B), EGF (PeproTech, 100-15) and PDGF-aa (Peprotech, 100-13A-10), and PDGF-aa (Peprotech, 100-13A-10), propidium iodide (PI) from Invitrogen and Allophycocyanin (APC) Annexin V from BD Pharmingen 550474 BD).

### Human neural stem cell culture

Human brain embryonic tissue was provided by the Human Developmental Biology Resource (HDBR, http://www.hdbr.org/). All procedures using human tissue were carried out in accordance with the Human Tissue Act 2006 with informed consent (REC reference: 18/LO/0822) for study participation under ethical approval (NRES Committee London – Fulham, London, UK). The hNSC lines used in this study had been derived from embryonic brain tissue at Carnegie Stage (Cs)17 and Cs23, and grown on laminin, as previously described ( Taylor *et al*., 2019; U *et al*., 2014; Vagaska *et al*., 2016). In brief, cells were seeded at a density of ~11,000 cells/cm ^2^ and grown at 37°C in a humidified incubator with 5% CO _2_ in medium containing: Dulbecco's Modified Eagle Medium/Nutrient Mixture F-12 (DMEM/F12) supplemented with 1% (v/v) penicillin/streptomycin streptomycin (Life Technologies, 15140122), 1% (v/v) 100x N2, 2% (v/v) B27, 20 ng/ml FGF-2, 20 ng/ml EGF, 50 µg/ml, BSA fraction V (85040C), 5 µg/ml (H3149) heparin and 10 µg/ml laminin (L2020).

### Differentiation protocols

Differentiation was induced when hNSCs had reached confluency, approximately 3 days after plating.

*Neuronal differentiation.* After 10 days in a medium consisting of DMEM containing Glutamax supplemented with 1% penicillin/streptomycin (F3917), 10 µM forskolin, 5 mM KCl, 2 mM valproic acid (P4543), 1 µM hydrocortisone and 5 µg/ml insulin (I9278) for 10 days, cells were maintained in with Neurobasal®-A Medium supplemented with 1% L-glutamine (Thermo Fisher Scientific, 25030-024), 1% penicillin/streptomycin and 2% B27 for 18 days (4 weeks total differentiation time). Protocol adapted from Guasti *et al*. (2012).

*Oligodendrocyte differentiation.* hNSCs were first incubated in DMEM/F12 containing 1% penicillin/streptomycin, 1% N2, 10 nM forskolin, 10 ng/ml FGF-2 and 10 ng/ml PDGF-aa for 14 days, and then in DMEM/F12 medium supplemented with 1% penicillin/streptomycin, 1% N2, 30 ng/ml tri-iodothyronine (T6397), 200 µM ascorbic acid and 10 ng/ml PDGF-aa for 7 days. PDGF-aa was then removed and cell incubated for a further 2 weeks to allow maturation (5 weeks total differentiation time).

*Astrocytic differentiation.* This was induced by incubating hNSCs in DMEM/F12 supplemented with 10% (v/v) FBS and 1% penicillin/streptomycin for 2 weeks.

### BES configuration and cell preparation

The BES system consisted of a high-voltage power supply (Glassman Europe Ltd., FP-30, Tadley, UK.) with a syringe pump (Harvard Apparatus) holding a needle similar to those used in our previous studies ( O’Neill *et al.*, 2019), which is a standard straight cut hypodermic stainless steel needle with 1.5-mm outer diameter and 0.8–0.9 mm inner diameter. The field strength was 0.2kv/mm (10kv over 50mm). The voltage was set at 10 kV and the flow rate at 250 ml/h. Because of the high flow rate, cells were subjected to the voltage for less than a minute. The procedure was carried out inside a class II biosafety cabinet to ensure sterility. hNSC suspensions with a density of ~1.3×10 ^6^ cells per ml were divided into 1 experimental and 2 control groups, all run in triplicate. The experimental hNSCs were taken to the the bio-electrospray facility, which was located in a different building, and sprayed (BES group). One control group was transported to the BES facility but not sprayed (TC), and the other was left in the tissue culture laboratory (LC). All groups were replated at the same time.

### Analysis of cell death and survival

The live/dead staining was performed 24 hours after BES. Hoechst 33258 and propidium iodide dissolved in phosphate buffered saline (PBS) were added to the culture medium at final concentrations of 2 μg/ml and 5 μg/ml, respectively. After a 2-hour incubation, cells were viewed and imaged using an IX71inverted microscope from Olympus equipped with a Lumen 200 metal arc lamp (Prior Scientific) and a monochrome ORCA-R2 digital camera (Hamamatsu Corp.) All images were processed with Fiji software (Java 8 version) ( Schindelin *et al*., 2012).

Cells viability/metabolic activity was assessed 1, 3 and 6 days after BES by the MTT (3-(4, 5-dimethylthiazolyl-2)-2, 5-diphenyltetrazolium bromide) assay. In brief, cells were incubated for 2 hours in medium containing 10% MTT (stock solution 5 mg/ml in DMSO), after which the absorbance was measured at 595 nm with a spectrophotometer (Multiscan FC ThermoScientific).

Flow cytometry analysis was performed immediately after and 3 days after BES. Roughly 1×10 ^6^ hNSCs per sample were resuspended in 500 µl of 1:100 APC-Annexin V conjugate:Annexin V binding buffer (10 mM HEPES, 150 mM NaCl, 5 mM KCl, 5 mM MgCl _2_ and 1.8 mM CaCl _2_ adjusted with NaOH to pH 7.4). Samples were kept at room temperature (RT) in the dark for 20 minutes before adding propidium iodide (PI) to a final concentration of 5 µg/ml. Stained cells were kept on ice until loading on a BD FACSCalibur TM to carry out flow cytometry analysis. Data was analysed using Kaluza 1.3 software. As a positive control, hNSCs were treated with 10 µm thapsigargin (T9033) for 24 hours to induce cell death prior to flow cytometry.

### Immunocytochemistry

Cells were fixed with 4% (w/v) paraformaldehyde (PFA) pH 7.4 for 15 minutes at RT, rinsed in PBS (phosphate buffer saline) and incubated in blocking solution (10% FBS, 3% BSA and 0.2% TritonX-100 in PBS) for 1 hour at RT. Incubation with primary and secondary antibodies at the indicated dilutions ( Table 1) was overnight at 4°C, and for 1 hour at RT, respectively. The nuclear stain Hoechst 33258 (2 μg/ml) was added to the secondary antibody solution. Cells were mounted with Citifluor (Citifluor Ltd). An IX71inverted microscope (Olympus) with a monochrome ORCA-R2 digital camera (Hamamatsu Corp.) was used to acquire images. All images were processed with Fiji software ( Schindelin *et al*., 2012).

**Table 1.** Primary and secondary antibodies used for immunofluorescence.

Primary Antibodies					
*Target*	*Antibody type (species)*	*Supplier*	*Product* *Number*	*Dilution*	*RRID*
NeuN	Monoclonal (mouse)	Millipore	MAB377	1/100	AB_2298772
NF200	Polyclonal (rabbit)	Sigma	N4142	1/100	AB_477272
S100β	Polyclonal (rabbit)	Dako	Z0311	1/100	AB_10013383
DCX	Polyclonal (rabbit)	Invitrogen	48-1200	1/200	AB_2533840
MAP2	Monoclonal (mouse)	Life Technologies	131500	1/200	AB_2533001
GFAP	Polyclonal (rabbit)	Millipore	AB5804	1/500	AB_2109645
Vimentin	Monoclonal (mouse)	Dako	GA630	1/500	AB_2827759
O4	Monoclonal (mouse)	R&D	MAB1326	1/100	AB_357617
A2B5	Monoclonal (mouse)	R&D	MAB1416	1/100	AB_357687
Secondary Antibodies					
*Target*	*Host (fluorophore)*	*Supplier*	*Product* *Number*	*Dilution*	*RRID*
Mouse IgG	Donkey (Alexa Fluor 488)	Molecular Probes	A-21202	1/500	AB_141607
Rabbit IgG	Donkey (Alexa Fluor 594)	Molecular Probes	A-21207	1/500	AB_141637
Mouse IgM	Goat (Alexa Fluor 488)	Molecular Probes	A-21042	1/500	AB_2535711

### RNA analysis by reverse transcription-PCR (RT-PCR)

RNA was extracted from cell pellets using RNeasy Mini Kit (Qiagen) according to the manufacturer’s protocol. Complementary DNA (cDNA) was prepared from 500 ng of extracted RNA using MMLV reverse transcriptase (Promega, M1705) following the manufacturers protocol. Reverse transcription reactions were performed using a PTC-100 thermal cycler (MJ Research, Inc.). The sequences of the primers and conditions used are shown in Table 2. PCR reactions were performed in a Veriti Thermal Cycler (Applied Biosciences). To exclude contamination of the reagents, no-template controls (NTC) where water instead of cDNA was included were run in each experiment. A cDNA sample from a human embryonic brain (22 weeks post conception) was used as positive controls. Amplified products were separated by gel electrophoresis using 1.5% (w/v) agarose gels in tri-acetate EDTA (TAE) buffer and 1X SYBR Safe dye (ThermoFisher Scientific). Semi-quantification of the bands was performed using Fiji software ( Schindelin *et al*., 2012) and the housekeeping gene, glyceraldehyde 3-phosphate dehydrogenase (GAPDH), used to normalize expression.

**Table 2.** List of human primers and conditions used for transcript amplification.

Gene	Primer Sequences	Annealing temperature (°C)	Cycle N°
**GAPDH**	**Fw** CCTTCATTGACCTCAACTACATGGT **Rv** CTAAGCAGTTGGTGGTGCAGGA	56	30
**MAP2**	**Fw** CCACCTGAGATTAAGGATCA **Rv** GGCTTACTTTGCTTCTCTGA	59	30
**OLIG2**	**Fw** CAGAAGCGCTGATGGTCATA **Rv** TCGGCAGTTTTGGGTTATTC	56	32
**GFAP**	**Fw** GAAGCTCCAGGATGAAACCA **Rv** ACCTCCTCCTCGTGGATCTT	56	30
**NEFH (NF-H)**	**Fw** TAGCCGCTTACAGAAAACTC **Rv** AGACTTCTCCACCACTTTGA	56	32
**SLC1A3** **(GLAST)**	**Fw** CTCACAGTCACCGCTGTCAT **Rv** CCATCTTCCCTGATGCCTTA	56	32
**ENO2 (NSE)**	**Fw** CTGATGCTGGAGTTGGATGG **Rv** CCATTGATCACGTTGAAGGC	56	32

### Statistical analysis

Each experiment was performed in biological triplicates unless stated otherwise. Statistical analysis was carried out with two-way ANOVA followed by Tukey’s multiple comparison test. Results are expressed as mean ± standard error of the mean. Differences were considered to be significant if p ≤ 0.05.

## Results

### Assessment of hNSC viability after BES

We first investigated whether hNSC viability is affected immediately after and at different times after bioelectrospraying (BES). Three groups were compared in all experiments: bio-electrosprayed hNSCs (BES) and two control groups, the BES control hNSCs (cells transported to the BES laboratory, but not sprayed; TC) and the tissue culture laboratory control hNSCs (cells not transported to the BES laboratory; LC).

**Figure 1.**
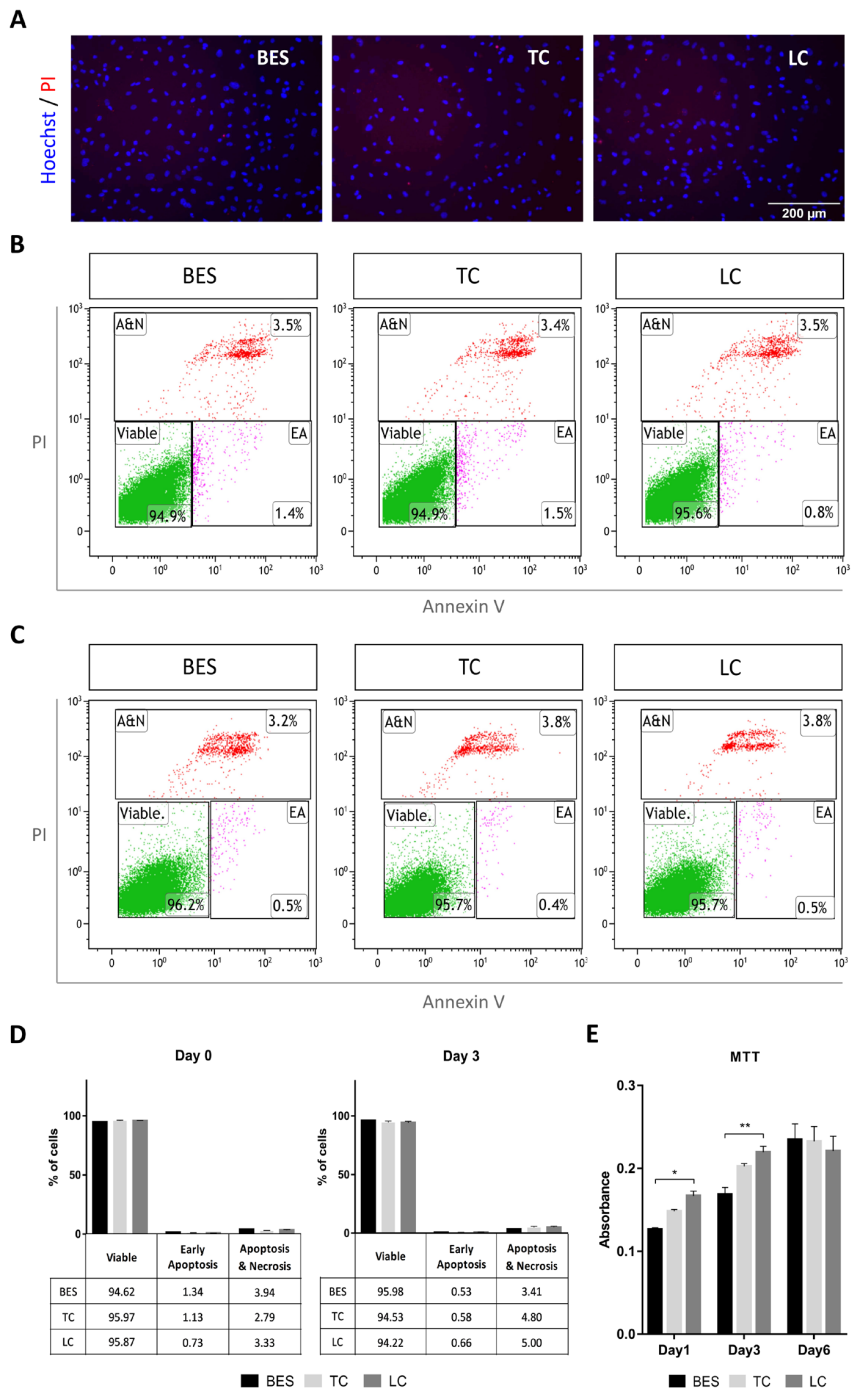
Viability of human neural stem cells (hNSCs) after bio-electrospray. **A**) Staining with propidium iodide (PI, red) and Hoechst 33258 (blue) in live hNSCs (Cs 17, passage 22) 24 hours after spraying (BES) and in non-sprayed controls (TC: taken to the BES laboratory but non-sprayed; LC: not moved from the tissue culture laboratory). All pictures are at the same magnification. **B**– **C**) Analysis of Annexin V- and PI- positive cells by flow cytometry immediately after spraying ( **B**) and 3 days after spraying ( **C**). Representative scatter plots showing early apoptotic (EA), and apoptotic plus necrotic cell population (A&N) measured as percentages of total gated cells. **D**) Cell populations represented as percentages of total gated cells. No significant difference in the percentage of viable cells is observed between BES and controls (biological triplicates presented as mean ± SEM) as assessed by two way ANOVA. **E**) hNSC metabolic activity assessed by the MTT assay 1, 3 and 6 days after BES. Data represent mean absorbance ±SEM, n=6; * p ≤ 0.05, ** p ≤ 0.01 as assessed by two way ANOVA and Tukey’s multiple comparisons test.

At 24 hours after spraying, cells were double stained with Hoechst dye and PI to detect dead cells. No apparent difference in cell death between BES and control groups was observed by cell imaging ( Figure 1A). To further assess the effect of BES on cell viability, hNSCs were labelled with PI and annexin V, a marker of apoptosis, immediately after spraying ( Figure 1B) and 3 days later ( Figure 1C). Cells were analysed by flow cytometry to detect early apoptotic (low PI and high APC-Annexin V), and late apoptotic/necrotic cells (high PI) as shown in Figure 1B, C. As summarized in Figure 1D, over 94% of cells were viable in all groups immediately after spraying (Day 0), and over 95% were viable at 3 days, with low levels of early apoptosis detected at both time points (1% and 0.5%, respectively). To establish whether BES affected cell behaviour over time, their metabolic activity, that reflects number of cells in the culture, was assessed by the MTT assay on day 1, 3 and 6 after BES. Metabolic activity increased over six days in all samples, although it was lower in the BES group than in the laboratory control at 1 and 3 days; however, by 6 days, no difference was observed among control and BES groups ( Figure 1E). Together, these results suggest that BES does not negatively affect hNSCs viability over time.

### Assessment of hNSCs differentiation potential after BES

**Figure 2.**
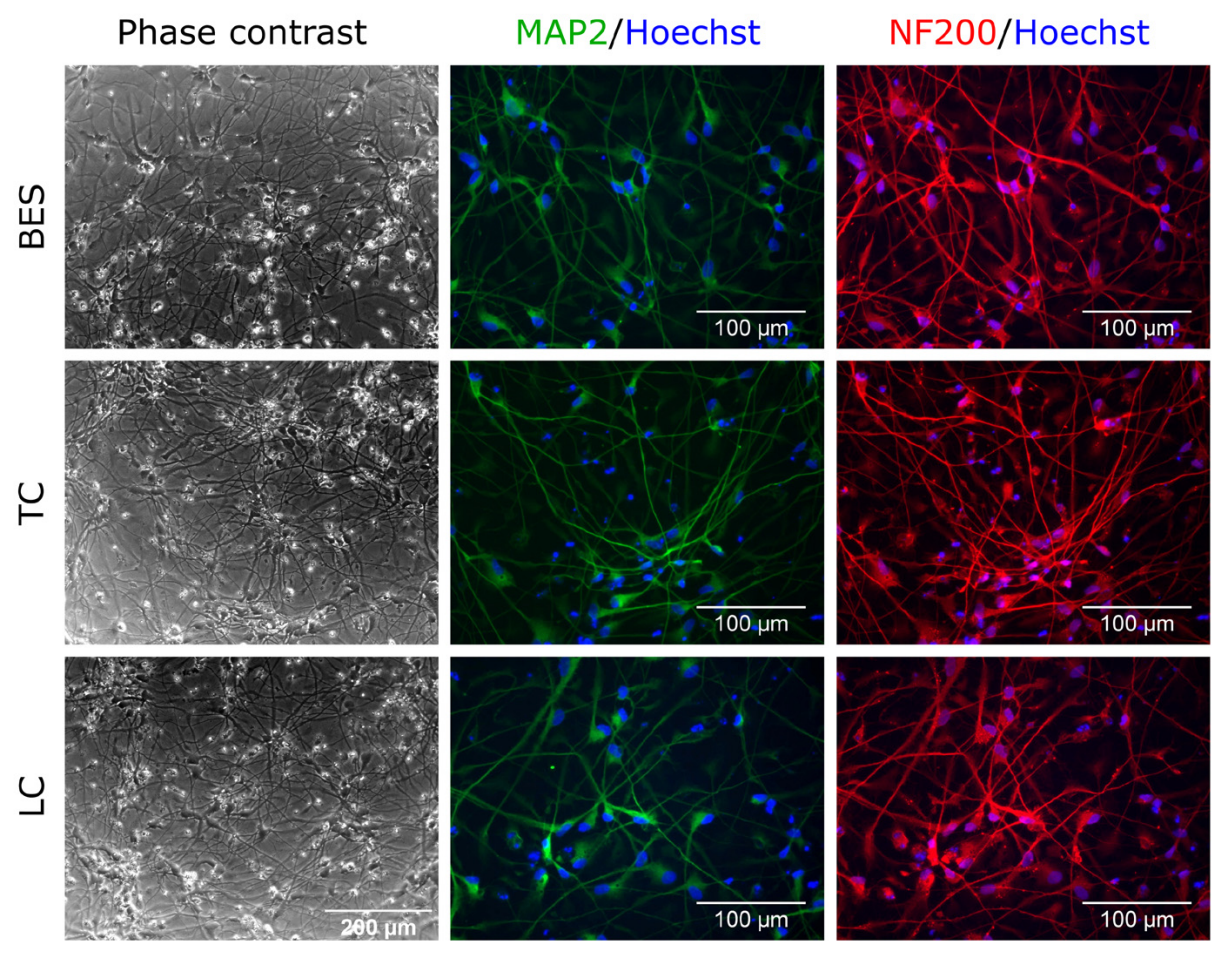
Neuronal differentiation of hNSCs after bio-electrospray assessed by phase contrast imaging and double-labelling for neuronal markers. Sprayed (BES) and control (TC: taken to the BES laboratory but non-sprayed; LC: not moved from the tissue culture laboratory) hNSCs (Cs 17, passage 22) differentiated for 4 weeks. Note typical neuronal morphology and neurite extension and expression of the neuronal markers, MAP2, microtubule-associated protein 2 (green), and NF200, neurofilament 200 (red). Nuclei are counterstained with Hoechst 33258 (blue). All phase contrast images are at the same magnification.

To establish whether BES affected hNSCs differentiation capacity, two hNSCs lines were differentiated along the neuronal and glial lineages (astrocytes and oligodendrocytes). After 4 weeks of neuronal differentiation, processes that had started to grow at 10 days (see Extended data) had extended further in all groups, as shown by phase contrast images ( Figure 2 and *Extended data*, Supplementary Figure S2) ( Ferretti & Helenes González, 2020b). Immunofluorescence labelling for neuronal markers revealed comparable expression of the mature neuronal markers microtubule-associated protein 2 (MAP2), neurofilament 200 (NF200) and neuronal nuclear protein (NeuN) ( Figure 2 and *Extended data*, Supplementary Figure S1 and S2) ( Ferretti & Helenes González, 2020b) in the control and BES groups. In contrast, no significant expression of doublecortin (DCX), a marker of newly born and migrating neurons was detected in any group ( *Extended data*, Supplementary Figure S1) ( Ferretti & Helenes González, 2020b). These indicated that the BES process did not compromise hNSC neuronal differentiation.

**Figure 3.**
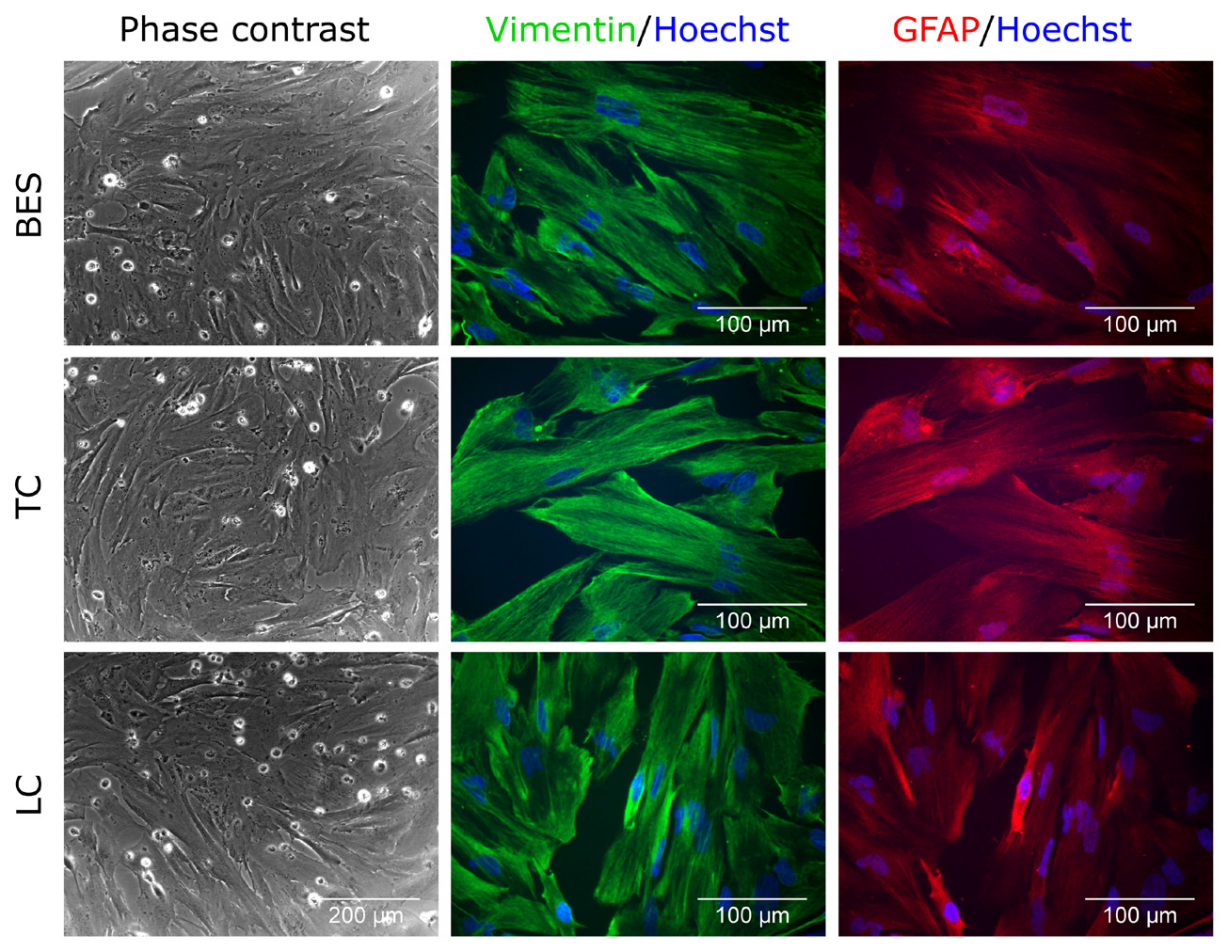
Astrocyte differentiation of hNSCs after bio-electrospray assessed by phase contrast imaging and double-labelling for astrocyte markers. Sprayed (BES) and control (TC: taken to the BES laboratory but non-sprayed; LC: not moved from the tissue culture laboratory) hNSCs (Cs 17, passage 22) differentiated for 2 weeks. Note the flatten morphology typical of astrocytes morphology and expression of astrocyte markers, Vimentin (green) and GFAP (glial fibrillary acidic protein; red). Nuclei are counterstained with Hoechst 33258 (blue). All phase contrast images are at the same magnification.

Upon induction of astrocyte differentiation, hNSC morphology rapidly changed in both control and BES groups, with cells becoming more spread and flatter than in undifferentiated controls, consistent with astrocytic differentiation ( Figure 3 and *Extended data*, Supplementary Figure S3) ( Ferretti & Helenes Gonzalez, 2020b). This was further supported by expression of vimentin and glial fibrillary acidic protein (GFAP). Together, comparable morphological appearance and glial markers expression in all groups indicates that BES does not interfere with astrocytic differentiation of hNSCs.

**Figure 4.**
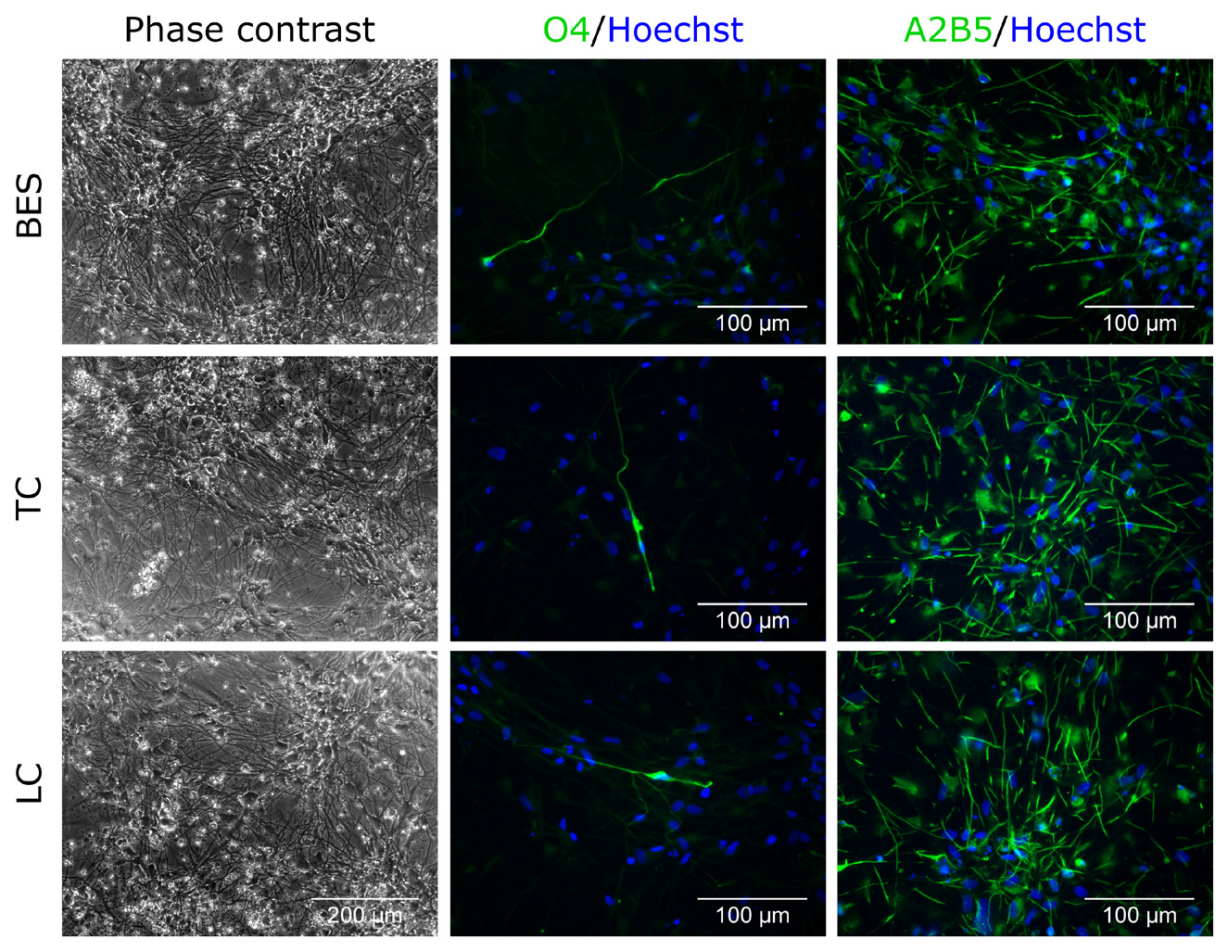
Oligodendrocyte differentiation of hNSC assessed after bio-electrospray by phase contrast imaging and immunostaining. Sprayed (BES) and control (TC: taken to the BES laboratory but non-sprayed; LC: not moved from the tissue culture laboratory) hNSCs (Cs 17, passage 22) differentiated for 5 weeks. Note the presence of cells with different morphologies with a few expressing the oligodendrocyte marker, O4, and a larger proportion the glial precursor marker, A2B5. Nuclei are counterstained with Hoechst 33258 (blue). All phase contrast images are the same magnification.

Finally, the effect of BES on oligodendrocyte differentiation was tested. At 5 weeks of differentiation after BES, both control and BES cultures had acquired a branched morphology with long processes ( Figure 4 and *Extended data*, Supplementary Figure S4) ( Ferretti & Helenes González, 2020b). Labelling of oligodendrocyte progenitor markers revealed a few cells positive for the oligodendrocyte marker, O4 ( Figure 4 and *Extended data*, Supplementary Figure S4) ( Ferretti & Helenes González, 2020b), and several positive for A2B5, that is expressed in oligodendrocyte progenitor cells ( Figure 4). There was no visible difference in the expression of these markers between BES cells and their controls, indicating that the BES process does not alter the oligodendrocyte differentiation.

### Effect of BES on gene expression in neuronally differentiated hNSCs

**Figure 5.**
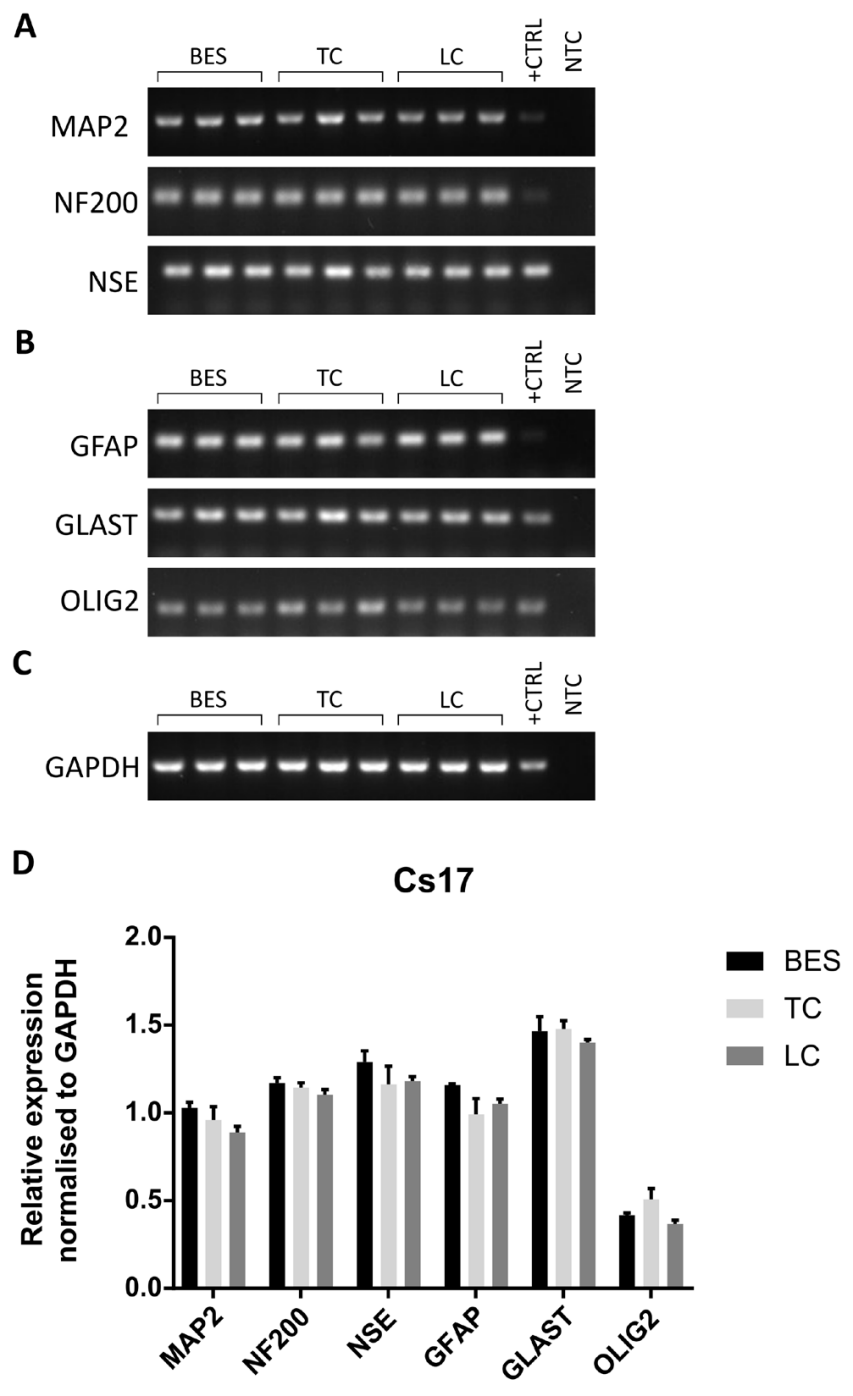
Expression of neural markers in hNSCs neuronally differentiated for 4 weeks after bio-electrospray assessed by RT-PCR. **A**– **C**. Expression of neuronal markers ( **A**), glial markers ( **B**), and a reference house-keeping gene, GAPDH, ( **C**) in biological triplicates of sprayed (BES) and control (TC: taken to the BES laboratory but non-sprayed; LC: not moved from the tissue culture laboratory) hNSCs (Cs 17, passage 25). MAP2: microtubule-associated protein 2; NF200: neurofilament 200; NSE: neuron-specific enolase; GFAP: glial fibrillary acidic protein; GLAST: glutamate aspartate transporter; OLIG2: oligodendrocyte transcription factor 2; GAPDH: glyceraldehyde 3-phosphate dehydrogenase. +CTRL: human embryonic brain cDNA used as positive control; NTC: no template control using water instead of cDNA. **D**) Relative expression of neuronal and glial markers assessed by densitometry. Data are means ± SEM of band intensity normalised to GAPDH. No significant difference in gene expression is observed (two way ANOVA).

The effect of BES on neuronal differentiation was further investigated at the gene expression level in hNSCs after 4 weeks of neuronal differentiation. As mixed cultures are normally obtained following neuronal induction, rather than pure neuronal populations, both neuronal and glial markers were assessed to establish whether BES changed the balance of differentiation among these cell types. Transcripts for the neuronal markers, NF200, neuron-specific enolase (NSE) and MAP2, as well as the glial markers glutamate aspartate transporter (GLAST), Olig2 and GFAP were detected in all groups by RT-PCR ( Figure 5A–C and *Extended data*, Supplementary Figure S5A–C) ( Ferretti & Helenes González, 2020b). To obtain more quantitative information, gene expression was further investigated by semi-quantitative RT-PCR analysis in two hNSC lines ( Figure 5D and *Extended data*, Supplementary Figure S5D) ( Ferretti & Helenes González, 2020b). Relative expression of genes was normalised to GAPDH. No significant differences in glial marker expression between the control and BES groups was observed in either line. Also, neuronal markers were expressed at comparable levels, with the exception of NF200, which was expressed at slightly higher levels in the BES group in one of the cell lines ( *Extended data*, Supplementary Figure S5D) ( Ferretti & Helenes González, 2020b). Overall, the expression of all but one marker were unchanged, suggesting no significant effects resulted from BES.

## Discussion

Only a few studies on the effect of BES on human cells have been carried out; these focussed on human mesenchymal stem cells (MSCs), either primary or hTERT immortalized, and tumour cells ( Braghirolli *et al*., 2013; Eddaoudi *et al*., 2010; Mongkoldhumrongkul *et al*., 2009a; Ye *et al*., 2015). In this study, to our knowledge we show for the first time that hNSCs can withstand the BES procedure without any negative effect on their self-renewal capacity and importantly on their neuronal and glial differentiation potential. The high voltage of 10kV used here, the pressure applied by the syringe pump, the flow rate, the high-density solution in a small-bore needle (0.8–0.9 mm) and the handling of the cells in a separate laboratory had limited impact on hNSCs. It is well established that high voltages can be detrimental to cells, for example when cells are electroporated ( Traitcheva & Berg, 2010). However, the fact that BES operates at high voltage but low current, in the nano-ampere range ( Pakes *et al*., 2011; Poncelet *et al*., 2012), could help explain why cells do not show adverse effects when subjected to high voltages. Recently, a study assessing the effect of bioprinting on Schwann cells and myoblasts has suggested that this technique affects their viability and proliferative activity ( Ning *et al*., 2018). Therefore, at least for some cell types, BES could provide a valuable alternative to bioprinting.

### hNSCs viability is not affected by BES

Our findings on the safety of BES on hNSCs are consistent with findings in a number of cell types and organisms previously investigated, including mesenchymal cells ( Hong *et al*., 2010; Irvine *et al*., 2007; Jayasinghe *et al*., 2011; Ye *et al*., 2015), immortalised mouse neural cells and human astrocytoma cells ( Eagles *et al*., 2006; Eddaoudi *et al*., 2010; Jayasinghe & Townsend-Nicholson, 2006), and nematodes ( Mongkoldhumrongkul *et al*., 2010). In all these studies, a survival of up to 90% after BES was observed. Notably, the hNSCs displayed an even higher survival rate (>94%), suggesting that these human stem cells are more robust than most of the cells previously studied.

Another difference between previous studies and ours is the length of time we monitored the cells for. Here, not only we measured metabolic activity up to 6 days, but also monitored the cells over weeks in the differentiation experiments, where undifferentiated controls were run in parallel. The use of two controls also showed that an initial small decrease in metabolic activity on BES samples was partly due to the transfer of the samples to the BES laboratory, and that full recovery had occurred by day 6. Viability of rabbit bone marrow-derived MSCs after BES was lower than that of hNSCs, with a metabolic/proliferation rate significantly lower than in controls even at a lower bio-electrospraying voltage than that used in our study ( Sahoo *et al*., 2010). Together, the combination of cell death and survival assays and long-term monitoring used here provides clear evidence that BES does not affect hNSCs viability either immediately or over time.

### hNSCs differentiation potential is maintained after BES

Our tri-lineage differentiation study has clearly shown no changes in hNSC differentiation potential after BES. As from previous reports, hNSC induction of neuronal differentiation resulted in a heterogeneous population of cells, and this was comparable across groups as shown by mRNA expression ( Glaser *et al*., 2007; Gu *et al*., 2016; Sun *et al*., 2008). Astrocytic differentiation resulted in a more homogeneous population and all cells expressed vimentin and GFAP, though at different extents. The low number of cells positive for the oligodendrocyte marker O4 upon induction of oligodendrocyte differentiation is comparable in control and BES groups, and consistent with the long time required for human oligodendrocyte maturation. Indeed, a marker of less mature cells, A2B5, was expressed in a much higher proportion of cells.

This again supports the view that hNSCs are very resistant to external stimuli. This may be a property of human stem cells or of neural stem cells, or both, and extensive investigation will be required to compare stem cell types across species to clarify this issue. Rabbit bone marrow-derived MSCs have been shown to maintain differentiation potential along three mesenchymal lineages after BES at a lower voltage than the one used here ( Sahoo *et al*., 2010). By contrast, human MSCs derived from human deciduous tooth pulp appear to better withstand BES even at higher voltage (15 kV), as well as maintain tri-lineage differentiation potential ( Braghirolli *et al*., 2013; Braghirolli *et al*., 2015). Also human adipose-derived MSCs (ADSCs) survived and differentiated efficiently after BES ( Ye *et al*., 2015).

## Conclusion

Analysis of cell viability, tri-lineage differentiation capacity and gene expression demonstrated that the BES process does not adversely affect hNSCs either in the short or long term. Notably, it highlighted the robustness of these human stem cells. In conclusion, this study shows that BES is a suitable tool for the direct handling of hNSCs. Therefore, it may provide a suitable technology for deposition of hNSCs to specific locations in damaged nervous system *in vivo* or within suitable scaffolds for neural tissue engineering. Furthermore, this approach could be developed to generate well-controlled human neural 3D models for studying neural development or disease and responses to putative novel therapeutic interventions.

## Data availability

### Underlying data

Harvard Dataverse: Bio-electrosprayed human neural stem cells are viable and maintain their differentiation potential- Underlying data of main figures. https://doi.org/10.7910/DVN/CAASEG ( Ferretti & Helenes González, 2020a).

This project contains the raw uncropped images used to produce each figure, in addition to flow cytometry, cell viability and RT-PCR output data.

Harvard Dataverse: Bio-electrosprayed human neural stem cells are viable and maintain their differentiation potential- Underlying data of supplementary figures. https://doi.org/10.7910/DVN/CLGEWR ( Ferretti, 2020).

This project contains the raw uncropped images used to produce each of the supplementary figures (see *Extended data*), in addition to RT-PCR output data for Supplementary Figure S5D.

### Extended data

Harvard Dataverse: Bio-electrosprayed human neural stem cells are viable and maintain their differentiation potential- Extended data. https://doi.org/10.7910/DVN/M8ZFNR ( Ferretti & Helenes González, 2020b).

This project contains the file ‘Supplementary figures.pdf’, which contains the following extended data:

- **Figure S1 Expression of neuronal markers in hNSCs after 4 weeks of differentiation.** (A–B) Neuronal nuclear protein (NeuN) and doublecortin (DCX) in sprayed (BES) and control (TC: taken to the BES laboratory but non-sprayed; LC: not moved from the tissue culture laboratory) in two hNSC lines, Cs 17, passage 22 (A) and Cs23, passage 20 (B). Nuclei are counterstained with Hoechst 33258 (blue).
- **Figure S2. Neuronal differentiation of hNSCs after bio-electrospray assessed by phase contrast imaging and double-labelling for neuronal markers.** Sprayed (BES) and control (TC: taken to the BES laboratory but non-sprayed; LC: not moved from the tissue culture laboratory) hNSCs (Cs 23, passage 20) differentiated for 4 weeks. Note typical neuronal morphology and neurite extension and expression of the neuronal markers, MAP2, microtubule-associated protein 2 (green), and NF200, neurofilament 200 (red). Nuclei are counterstained with Hoechst 33258 (blue). All phase contrast images are at the same magnification.
- **Figure S3. Astrocyte differentiation of hNSCs after bio-electrospray assessed by phase contrast imaging and double-labelling for astrocyte markers.** Sprayed (BES) and control (TC: taken to the BES laboratory but non-sprayed; LC: not moved from the tissue culture laboratory) hNSCs (Cs 23, passage 20) differentiated for 2 weeks. Note the flatten morphology typical of astrocytes morphology and expression of astrocyte markers, Vimentin (green) and GFAP (glial fibrillary acidic protein; red). Nuclei are counterstained with Hoechst 33258 (blue). All phase contrast images are at the same magnification.
- **Figure S4. Oligodendrocyte differentiation of hNSC assessed after bio-electrospray by phase contrast imaging and immunostaining.** Sprayed (BES) and control (TC: taken to the BES laboratory but non-sprayed; LC: not moved from the tissue culture laboratory) hNSCs (Cs 23, passage 20) differentiated for 5 weeks. Note the presence of cells with different morphologies with a few expressing the oligodendrocyte marker, O4, and a larger proportion the glial precursor marker, A2B5. Nuclei are counterstained with Hoechst 33258 (blue). All phase contrast images are the same magnification.
- **Figure S5. Expression of neural markers in hNSCs neuronally differentiated for 4 weeks after bio-electrospray assessed by RT-PCR.** (A-C) Expression of neuronal markers (A), glial markers (B), and a reference house-keeping gene (C) in biological triplicates of sprayed (BES) and control (TC: taken to the BES laboratory but non-sprayed; LC: not moved from the tissue culture laboratory) hNSCs (Cs 23, passage 22). MAP2: microtubule-associated protein 2; NF200: neurofilament 200; NSE: neuron-specific enolase; GFAP: glial fibrillary acidic protein; GLAST: glutamate aspartate transporter; OLIG2: oligodendrocyte transcription factor 2; GAPDH: glyceraldehyde 3-phosphate dehydrogenase. +CTRL: human embryonic brain cDNA used as positive control (22 weeks post conception); NTC: no template control using water instead of cDNA. (D) Relative expression of neuronal and glial markers assessed by densitometry. Data are means ± SEM of band intensity normalised to GAPDH. Increased NF200 expression (* *p* 0.05) is observed in the BES group (two way ANOVA with Tukey’s multiple comparisons test).

Data are available under the terms of the Creative Commons Zero “No rights reserved” data waiver (CC0 1.0 Public domain dedication).
