# Supplementary material for: Bio-electrosprayed human neural stem cells are viable and maintain their differentiation potential
Source: F1000Res. 2020 Jul 31;9:267. Originally published 2020 Apr 17. [Version 2] doi: 10.12688/f1000research.19901.2 (PMC7255967; doi:10.12688/f1000research.19901.2)
Supplement: Peer review history

# Peer review: Reviewer response for version 2

**DOI:** 10.5256/f1000research.27411.r68464

**Published:** 2020-08-18

**Authors:** Jingwei Xie

**Affiliations:** 1 Department of Surgery‐Transplant and Mary and Dick Holland Regenerative Medicine Program, College of Medicine, University of Nebraska Medical Center, Omaha, NE, USA

No further comments.

Is the work clearly and accurately presented and does it cite the current literature?

Yes

If applicable, is the statistical analysis and its interpretation appropriate?

Yes

Are all the source data underlying the results available to ensure full reproducibility?

No source data required

Is the study design appropriate and is the work technically sound?

Yes

Are the conclusions drawn adequately supported by the results?

Yes

Are sufficient details of methods and analysis provided to allow replication by others?

Yes

Reviewer Expertise:

Electrospray, Biomaterials, Tissue Regeneration

I confirm that I have read this submission and believe that I have an appropriate level of expertise to confirm that it is of an acceptable scientific standard.

# Peer review: Reviewer response for version 2

**DOI:** 10.5256/f1000research.27411.r68466

**Published:** 2020-08-03

**Authors:** Jamie A. Davies

**Affiliations:** 1 Deanery of Biomedical Sciences, University of Edinburgh, Edinburgh, UK

Thanks for the extra information, and congratulations on a useful article. JAD.

Is the work clearly and accurately presented and does it cite the current literature?

Yes

If applicable, is the statistical analysis and its interpretation appropriate?

Yes

Are all the source data underlying the results available to ensure full reproducibility?

Yes

Is the study design appropriate and is the work technically sound?

Yes

Are the conclusions drawn adequately supported by the results?

Yes

Are sufficient details of methods and analysis provided to allow replication by others?

No

Reviewer Expertise:

Tissue engineering & Synthetic Biology (but my reservations, which are purely about more methodological details being needed in places, do not depend on any deep expertise).

I confirm that I have read this submission and believe that I have an appropriate level of expertise to confirm that it is of an acceptable scientific standard.

# Peer review: Reviewer response for version 1

**DOI:** 10.5256/f1000research.21837.r62499

**Published:** 2020-05-22

**Authors:** Patricia Pranke

**Affiliations:** 1 Hematology and Stem Cell Laboratory, Faculty of Pharmacy, Universidade Federal do Rio Grande do Sul (UFRGS), Porto Alegre, Brazil

The article analyzes the effect of the bio-electrospray (BES) technique on human neural stem cells (hNSCs). Although the BES provoked a decrease in hNSC metabolic activity, there was no increase in hNSC death. The differentiated assays were also successful, showing that the BES did not adversely affect the differentiation capacity of the hNSCs or their gene expression.

 The figures are very illustrative and very well executed.

 Please correct VERY instead of “VEY well and could….” In the line 1 on the second column of the Introduction.

 The paper is very well written and it could probably be indexed without additional revision.

 Having said that, there are a few minor errors which could be adjusted, but they are small details.

 I have 6 more additional examples if you need them.

  In the Abstract:

 “Bio-electrospray (BES) is a jet based” – change to “Bio-electrospraying (BSE)”

 “…has the ability to form...” – “..has the ability of forming...”

 “...different hNSC lines..' – '...a number of hNSC lines...”

 “...from which cells recovered...” – “...from which the cells recovered...”

 “...as efficiently as controls…” – “...as efficiently as the controls…”

 “...and oligodentocytes as shown by...” – “...and oligodentrocytes, as shown by...”

Is the work clearly and accurately presented and does it cite the current literature?

Yes

If applicable, is the statistical analysis and its interpretation appropriate?

Yes

Are all the source data underlying the results available to ensure full reproducibility?

Yes

Is the study design appropriate and is the work technically sound?

Yes

Are the conclusions drawn adequately supported by the results?

Yes

Are sufficient details of methods and analysis provided to allow replication by others?

Yes

Reviewer Expertise:

Tissue engineering and regenerative medicine - area of stem cell and nanotechnology (electrospinning mainly)

I confirm that I have read this submission and believe that I have an appropriate level of expertise to confirm that it is of an acceptable scientific standard.

# Response

**Published:** 2020-06-15

**Authors:** Patrizia Ferretti

**Affiliations:** Institute of Child Health, UCL Great Ormond Street Institute of Child Health, UK

We thank you the Reviewer for their kind comments and useful suggestion which we have incorporated in the revised version of the manuscript.

# Peer review: Reviewer response for version 1

**DOI:** 10.5256/f1000research.21837.r62498

**Published:** 2020-05-11

**Authors:** Jingwei Xie

**Affiliations:** 1 Department of Surgery‐Transplant and Mary and Dick Holland Regenerative Medicine Program, College of Medicine, University of Nebraska Medical Center, Omaha, NE, USA

This work reports bioelectrospray of human neural stem cells and examines the survival, growth, and differentiation after bioelectrospray. This is an interesting work as bioelectrospray could be used to deposit human neural stem cells to form patterns for forming and studying complex neural tissue constructs. Overall, this work is satisfactory. However, authors should provide details of the bioelectrospray process. For example, how long for the process? In addition, the flow rate 250 ml/h seems too high. Please double check.

Is the work clearly and accurately presented and does it cite the current literature?

Yes

If applicable, is the statistical analysis and its interpretation appropriate?

Yes

Are all the source data underlying the results available to ensure full reproducibility?

No source data required

Is the study design appropriate and is the work technically sound?

Yes

Are the conclusions drawn adequately supported by the results?

Yes

Are sufficient details of methods and analysis provided to allow replication by others?

Yes

Reviewer Expertise:

Electrospray, Biomaterials, Tissue Regeneration

I confirm that I have read this submission and believe that I have an appropriate level of expertise to confirm that it is of an acceptable scientific standard, however I have significant reservations, as outlined above.

# Response

**Published:** 2020-06-15

**Authors:** Patrizia Ferretti

**Affiliations:** Institute of Child Health, UCL Great Ormond Street Institute of Child Health, UK

We thank the Reviewer for this comment. It should be noted that this study focused on the initial crucial question concerning cell viability and maintenance of differentiation capability after bioelectrospraying rather than on the formation of the stable cone jet mode needed to obtain micro to nano-sized droplets. Hence, a high flow rate was used to evaluate cell viability when exposed to the high voltage and needle constraints of BES. Because of the high flow rate, cells were subjected to the voltage for less than a minute. This study provides the foundation for the use of hNSCs to refine the conditions for achieving a stable cone jet mode. To this purpose, flow rate adjustments, single or coaxial needle arrangement, and liquid properties mentioned will need to be analysed.

# Peer review: Reviewer response for version 1

**DOI:** 10.5256/f1000research.21837.r62497

**Published:** 2020-05-01

**Authors:** Jamie A. Davies

**Affiliations:** 1 Deanery of Biomedical Sciences, University of Edinburgh, Edinburgh, UK

Overall, this study is a report of useful, incremental research. Previous reports, including some by the same lead author, have shown that a range of mammalian cells tolerate BES well. This report effectively says “and so do hNSCs”. Not a surprise (there was no obvious reason that the wouldn't) but a useful thing to know nevertheless.

 Specific comments:

 I liked your use of both transported and un-transported controls.

 In the methods, it would be helpful to have catalogue numbers of all of the biologics (growth factors etc), because these can be purchased in subtly different forms (native, recombinant, etc). Knowing the exact one helps the cause of reproducibility.

 In the first paragraph of the Introduction, 3 lines up, it would be helpful to know what BES conditions can be altered (otherwise it is hard to make the comparison with the less advantageous techniques mentioned in the subsequent sentence).

 Methods, 2 ^nd^ paragraph, last line – what is N2? If it is the culture supplement that ThermoFisher (and maybe others) sell, please give a reference or at least an explanation.

 Can you say more about the needle? (eg supplier code) and the field strength (you keep quoting 10kV, but not the distance over which this potential exists). Again, this is for reproducibility.

 It is also critical that you provide more information about the BES setup (distances etc); assuming this is one you have described in previous publications, you can just cite these publications. But without this information, nobody could reproduce this work.

 Strictly, the whole manuscript requires careful copy-editing to remove minor errors of punctuation and syntax, but these errors do not make the existing manuscript too hard to understand.

Is the work clearly and accurately presented and does it cite the current literature?

Yes

If applicable, is the statistical analysis and its interpretation appropriate?

Yes

Are all the source data underlying the results available to ensure full reproducibility?

Yes

Is the study design appropriate and is the work technically sound?

Yes

Are the conclusions drawn adequately supported by the results?

Yes

Are sufficient details of methods and analysis provided to allow replication by others?

No

Reviewer Expertise:

Tissue engineering & Synthetic Biology (but my reservations, which are purely about more methodological details being needed in places, do not depend on any deep expertise).

I confirm that I have read this submission and believe that I have an appropriate level of expertise to confirm that it is of an acceptable scientific standard, however I have significant reservations, as outlined above.

# Response

**Published:** 2020-06-15

**Authors:** Patrizia Ferretti

**Affiliations:** Institute of Child Health, UCL Great Ormond Street Institute of Child Health, UK

We are grateful to the Reviewer for his helpful comments. **“**In the methods, it would be helpful to have catalogue numbers of all of the biologics (growth factors etc), because these can be purchased in subtly different forms (native, recombinant, etc). Knowing the exact one helps the cause of reproducibility.”

This has been included, but please note that full information about a couple of reagents is still missing because we do not have access to it due to the current lockdown to control the Covid-19 pandemic.

“In the first paragraph of the Introduction, 3 lines up, it would be helpful to know what BES conditions can be altered (otherwise it is hard to make the comparison with the less advantageous techniques mentioned in the subsequent sentence).”

The configuration needed to obtain micro to nano-sized droplets, and a near mono-distribution can be achieved by adjusting BES conditions to obtain a stable cone jet mode. The primary conditions to consider are viscosity and electrical conductivity of the sprayed liquid. Ideally, the cell-laden suspension should have high viscosity and low electrical conductivity. A coaxial arrangement with an outer needle carrying an encapsulating biomaterial and an inner needle carrying the cells in suspension could also be considered (Jayasinghe and Townsend-Nicholson, 2006). This has now been specified.

 “Can you say more about the needle? (eg supplier code) and the field strength (you keep quoting 10kV, but not the distance over which this potential exists). Again, this is for reproducibility. It is also critical that you provide more information about the BES setup (distances etc); assuming this is one you have described in previous publications, you can just cite these publications. But without this information, nobody could reproduce this work.”

The needle used in these studies are similar to those used in our previous studies ^[x]^, and is a standard straight cut hypodermic needle made of stainless steel. The field strength was 0.2kv/mm (10kv over 50mm) and a reference has been added: O'Neill HC, Maalouf WE, Harper JC, Jayasinghe SN. Bio-electrosprayed human sperm remain viable. *Materials Today*. 2019; 1;31:21-30. **“**
